# Queen Charlotte's Lying-In Hospital

**Published:** 1919-10-18

**Authors:** 


### THE EDITOR'S LETTER-BOX.

[Correspondence on all subjects is invited, but we cannot in any way be responsible for the opinions expressed by our correspondents, who must give their name and address as a guarantee of good faith, but not necessarily for publication. Correspondents are reminded that brevity of style and conciseness of statement greatly facilitate early insertion.]

### QUEEN CHARLOTTE'S LYING-IN HOSPITAL.

### A PROTEST AGAINST MISS FOWLER'S STATEMENTS.

Further correspondence continues to reach us as to the conditions within Queen Charlotte's Hospital, including a protest against the statements made by Nurse Fowler in our issue of October 4, some of which are flatly contradicted and resented. What steps are the authorities of Queen Charlotte's Hospital taking to meet the demand which has arisen for a small commission of inquiry, competent and independent enough to examine and deal with the allegations concerning this lying-in hospital? It should be evident to the Governors and Committee of Queen Charlotte's Hospital that they owe it to the public, the patients, the nursing profession, and present and possible pupil midwives, as well as to their institution that such an inquiry should be promptly held. Further, that any further delay in its appointment can only have one result. It is felt to be essential in the interests involved that such an independent commission is called for, or failing action on the part of the hospital authorities, the Ministry of Health through its President, Dr. Addison, may use the weight of his authority to procure the appointment of such a commission, and get it to work without further avoidable delay. The number of fully-trained midwives available to-day is none too large, and anything which may tend to lessen it must be a matter of grave concern to the Ministry of Health in the efficient discharge of some of its most responsible duties to the public.—Ed. The Hospital.

